# Factors that challenge health for people involved in the compensation process following a motor vehicle crash: a longitudinal study

**DOI:** 10.1186/s12889-015-1694-5

**Published:** 2015-04-09

**Authors:** Nieke A Elbers, Arno J Akkermans, Keri Lockwood, Ashley Craig, Ian D Cameron

**Affiliations:** John Walsh Centre for Rehabilitation Research, University of Sydney, Kolling Institute of Medical Research, Royal North Shore Hospital, St Leonards, NSW 2065 Australia; Amsterdam Centre for Comprehensive Law, Faculty of Law, VU University, De Boelelaan 1105, 1081 HV Amsterdam, The Netherlands

**Keywords:** Claimants, Traffic crash, Mental health, Anxiety, Insurance companies, Secondary victimisation, Compensation, Injury

## Abstract

**Background:**

Motor vehicle crashes (MVC) are associated with diminished mental health, and furthermore, evidence suggests the process of claiming compensation following an MVC further increases distress and impedes recovery. However, further research is required on why the compensation process is stressful. The aim of the current study is twofold. The first is to investigate whether the interaction with the insurance agency is associated with anxiety. The second is to explore qualitatively aspects of dissatisfaction with the compensation process.

**Methods:**

Participants (N = 417) were injured people involved in a compensation scheme after a motor vehicle crash (MVC) in New South Wales, Australia. Interviews were conducted by phone at 2, 12 and 24 months after the MVC. A suite of measures were used including compensation related measures, pain catastrophising and the anxiety/depressed mood subscale of the EuroQol. The association between predictors and anxiety/depressed mood as the dependent variable were analysed using forward logistic regression analyses. The comments about dissatisfaction with the insurance company were analysed qualitatively.

**Results:**

The strongest predictor of mood status found was pain-related catastrophising, followed by dissatisfaction with the insurance company. Dissatisfaction was attributed to (1) lack of communication and lack of information, (2) delayed or denied payments of compensation, (3) slow treatment approval and discussions about causality, (4) too much complicated paperwork, and (5) discussions about who was at-fault.

**Conclusions:**

Factors were found that contribute to anxiety in the compensation process. The association between catastrophising and anxiety/depressive mood suggests it is worthwhile further investigating the role of negative cognitions in compensation processes. People who score highly on catastrophising after the MVC may benefit from early psychological interventions aiming at addressing negative cognitions. Another important stressor is the interaction with the insurance company. Stress is associated with problems of communication, medical treatment, and claim settlement. This study additionally draws attention to some under recognised problems such as delayed payments. Pro-active claims management could address some of the identified issues, which could improve health of injured people after a MVC.

## Background

Evidence suggests injured people who claim compensation after a motor vehicle crash (MVC) do not recover as well as people with similar injuries who do *not* claim compensation. There is an abundance of research supporting this view [1]. However, while there have been questions raised as to the quality of some of these studies [2], such as that results are confounded by self-selection of participants to the compensation or no-compensation group [3], the consistency of evidence cannot be ignored. Reviews have found negative associations for both physical and mental health [4,5]. Furthermore, the evidence for the negative influence of compensation has been collected from a range of countries worldwide, and has included both workers’ and MVC compensation schemes.

Even though most research has suggested a relationship exists between being involved in a compensation process and poorer health following an MVC, few studies have investigated reasons why. Some researchers have suggested that people who claim compensation may have different personal characteristics than those who do not claim, such as a worse pre-injury health status or a different personality, and that these characteristics could contribute to poorer recovery [5]. Others suggest the contribution of *secondary gain* or *accident neurosis,* which suggests that claimants do not recover because of a financial incentive not to get better as long as the process lasts [6]. Finally, people involved in a compensation process could be impeded by the stress surrounding the process, including the adversarial attitude of legal professionals *(secondary victimisation)* [7,8].

The current study further investigated the latter theory, that is, whether the compensation process is inherently stressful and therefore associated with poorer well-being. It considered the possible elements of the compensation process that increase stress and anxiety. Claim factors that have been found to be stressful are, among others: claim duration, involvement in legal disputes, and lawyer engagement [7,9]. Interestingly, the impact of the interaction with the insurance company, which could be considered to have the biggest effect on claimants’ well-being, has not been well investigated. To our knowledge, only two quantitative studies include the insurance company as a possible influencing factor on health and well-being [8,10]. They both concluded that the stressful interaction with insurance companies was the most important factor explaining elevated levels of anxiety in people who claimed compensation. Both studies recruited their participants from trauma hospitals, which implies that their participants are more severely injured than the general claimant population. Further research is needed to replicate this finding in the average motor vehicle compensation population. Certainly more research is required that investigates those aspects that are related to increased stress and anxiety.

To our knowledge, only two qualitative studies have investigated experiences of injured people with the insurance company [11,12]. It should be noted that there are a number of qualitative studies that address the interaction between injured people and insurance companies [13,14], but most examined injured workers in workers’ compensation schemes, a process likely to involve different issues than for people who are involved in a motor accident compensation scheme. The study by Murgatroyd et al. [11] and Gabbe et al. [12] are unique as they were concerned with the latter. Participants reported they found the claims process adversarial and stressful due to factors such as a lack of communication, problematic treatment approvals (e.g. it took weeks to approve treatment requests, and sometimes it was a fight to get approval), and negotiating settlement was gruelling (e.g. procrastinating for as long as possible to maximise financial hardship, pressuring claimants to settle for a lesser amount) [11]. Delays in receiving benefits resulted in stress and financial hardship, and it was difficult to navigate through the claims process and the paperwork [12]. While these findings are valuable and illustrative, the participants’ interaction with the insurance company was only a sub-element of the study.

In order to address this gap in knowledge, the aim of the current study was twofold. The first was to replicate the finding that the interaction with the insurance company is associated with elevated anxiety in a general claimant population. The predictor of primary interest is dissatisfaction with the claims management process by the insurance company. Also, to achieve this first aim, additional variables were included, such as pain-related catastrophising [15] (that is believing that something is far worse than it actually is) and various claim factors (e.g. type of insurance company, lawyer engagement, previous claim, and claim settlement). The second aim was to establish why the interaction with the insurance company is perceived as stressful. To answer this research question, we explored the participants’ dissatisfaction with the insurance company in a thematic fashion, using qualitative analytical techniques.

## Methods

### Participants

Persons who sustained injuries in an MVC in New South Wales (NSW) between March and December, 2010, were eligible to participate in the study. Potential participants were identified from the Personal Injury Register (PIR) database held by the NSW Motor Accidents Authority (MAA), the government regulator of companies providing third party motor vehicle crash insurance in NSW. Insurance companies must follow specified Claims Handling Guidelines that are provided by the MAA [16]. Persons were excluded if they sustained catastrophic injuries (severe brain injury, acute spinal cord injury, or injury requiring hospitalisation for more than 7 days), were less than 18 years of age, not residents of NSW, more than 3 months post-injury, or unable to complete questionnaires by telephone in English [17].

A letter of invitation was sent by the MAA on behalf of the researchers together with a Participant Information Sheet. An opportunity to ‘opt out’ of the study was provided. Potential participants were then contacted by telephone approximately 2 weeks later. Participants were contacted until the research staff member’s time available for the month was exhausted. If verbal consent was given, the participant was invited to complete the initial interview by telephone approximately 2 months after the injury. Follow-up interviews were conducted at 12 and 24 months after injury. Interviews were conducted by an experienced research nurse [KL] acting independently from the MAA. The study was approved by the Ethics Committee of The University of Sydney and the study was conducted in accord with ethical guidelines for human research.

### Compensation setting

In New South Wales Australia compensation following motor vehicle crashes is available under a third party insurance scheme. This insurance is compulsory for the owner of all motor vehicles. People are eligible to lodge a claim if they are injured as a result of a motor vehicle accident. Damages up to $5,000 can be claimed under the Accident Notification Form regardless of who was at fault in the crash. For claims over $5,000 people need to lodge a personal injury claim, for which somebody else’s insurance is liable. Compensation can be paid for economic loss (lost wages and for past and future economic loss; loss of income is paid as lump sum at claim settlement), non-economic loss (pain and suffering and loss of quality of life if there is significant permanent impairment) and medical and rehabilitation costs. The Accident Notification Form needs to be sent to the insurer within 28 days of the accident. The fault-based personal injury claim should be lodged within 6 months post-accident [18].

### Measurements

#### Demographic variables

Data with reference to age, gender, country of birth, education, socio-economic status, and work status before the MVC were collected. Education was classified into low (primary education), medium (secondary education, certificate, advanced diploma), and high (bachelor degree, graduate diploma, postgraduate degree). Socio-economic status was represented by the Index of Relative Socio-Economic Disadvantage (IRSD) state decile score, obtained by matching participants’ post code to a Census Collection District [19]. The IRSD ranged from 1 to 10 (most disadvantaged – most advantaged). Work status options consisted of: employed, self-employed, unemployed, voluntary work, home duties, student and retired.

#### Injury variables

Pre-injury health was a self-rated measure (poor, fair, good, very good, excellent). Pain catastrophising was measured at all measurement points by means of the 9-item catastrophising subscale of the Pain-Related Self-Statements Scale (PRSS). This psychometric test has demonstrated reliability and validity [15]. Answer categories ranged from 0 to 5 (not at all – all the time) [15]. Examples of the questions are: ‘No matter what I do my pain doesn’t change’, ‘I am a hopeless case’. Higher scores indicate more frequent catastrophising. The sum is calculated and the total score range is 0–45.

Other injury data concerned injury severity and type of injury. Injury severity was defined by the New Injury Severity Score (NISS), which takes values from 0 to 75: mild (NISS 1–3), moderate (NISS 4–8), serious (NISS 9–15), severe (NISS 16–24), and critical (NISS 25–75) [20]. The NISS is derived from the Abbreviated Injury Scale [21], which is included in the MAA Personal Injury Registry (PIR) database. Type of injury consisted of three categories: whiplash, fractures, and other. As a component of their usual practice trained and experienced insurance company staff coded the reported injuries.

#### Compensation factors

Claim data included whether the participant had lodged a previous claim (yes/no), which insurance company dealt with the claim, whether they engaged a lawyer (yes/no, recorded at 12 and 24 months), claim settlement (yes/no, calculated by subtracting the crash date from the claim settlement date, dates were derived from the PIR database), and dissatisfaction with the claims management and/or the insurance company (a 6-point Likert scale at baseline [strongly disagree - strongly agree] and a 5-point Likert scale at 12 and 24 months [very unsatisfied - very satisfied]). The interview also contained an open answer question, in which participants were asked to explain their satisfaction or dissatisfaction with the claims management process. The interviewer summarised the participants’ responses in an Access database.

#### Quality of life and anxiety

Quality of life was measured by the EuroQol [22], consisting of five scales (i.e. mobility, self-care, usual activities, pain/discomfort, and anxiety/depressive mood) with a three point answer scale (no problems, some problems, or extreme problems) and a visual analogue scale (VAS) in which respondents indicated their health state for that day on a scale ranging from 0 to 100. The anxiety/depressive mood subscale of the EuroQol was used as the dependent measure in the regression analyses (1: no anxiety/depressive mood, 2: moderate anxiety/depressive mood, and 3: extreme anxiety/depressive mood).

### Quantitative analysis

Given there is scarce research that has investigated the impact of claim factors on health, the association between the independent variables and the dependent measure (i.e. anxiety/depressive mood) was calculated by means of multiple forward stepwise logistic regression analyses. A stepwise method was chosen, because this method is similar to the analysis conducted by O’Donnell et al [10]. Most variables were dichotomised. Socioeconomic status: lower (1–5) vs. higher (6–10). Education: low/medium vs. high. Work status: employed vs. unemployed. Pre-injury health status: poor-fair vs. good-excellent. Catastrophising: low (0–22) versus high (23–45; dichotomisation based on median). Type of injury: whiplash vs. other. Insurance company: one company representing 54% of participants vs. the five other companies; Claims management satisfaction: dissatisfied vs. satisfied. Anxiety/depressive mood: no anxiety/depressive mood versus moderate to extreme presence of anxiety/depressive mood. Three multiple regression analyses were conducted: one for each longitudinal measurement point (2, 12 and 24 months). The factors that were significantly associated with anxiety were determined (entry p = .05; removal p = .10). The analyses were conducted with SPSS version 21.

### Qualitative analysis

The qualitative analysis involved exploring the open answer satisfaction question. A grounded theory approach was used [23], consisting of labelling and categorising the comments of unsatisfied participants across the three measurements (2, 12 and 24 months after MVC). Analysis was conducted according to a cyclic process of open, axial and selective coding [23]. In the open coding phase, the transcripts were labelled with four keywords. The first three labels were ‘liability assessment’, ‘medical treatment and assessment’, and ‘determining compensation’, which were chosen because they indicate the chronological order of the claims process. These are phases that most claimants usually complete. The fourth keyword was ‘communication’, as this has been addressed in the literature as being a debilitating factor [2,11]. In the axial coding process, it was determined whether the labels needed to be restructured, whether sub-labels could be applied, and whether new labels emerged. In this phase, a fifth keyword called ‘paperwork’ was added, because claimants indicated that this was a problematic issue. During the selective coding, all the transcripts were re-analysed based on the refinement that occurred during axial coding. Furthermore, labels were structured in order of importance. The interviews were analysed in duplicate by two researchers [NE and IC]. During the cyclic analysis process, the two discussed their findings and, through discussion and negotiation, they agreed upon the final set of labels.

## Results

### Participants

**Figure 1:**
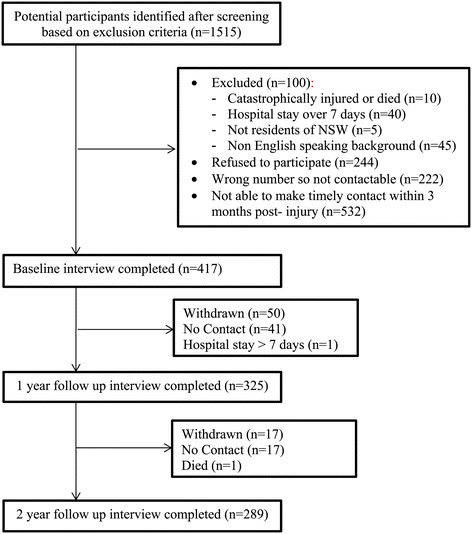
**Flowchart for participation in the study.**

A flowchart of progression of participants through the study is provided in Figure 1. A total of 1,515 insurance claims were identified after screening based on the exclusion criteria, of which 761 people were contacted to participate in the study. In total, another 100 people were excluded because they did not fulfil the inclusion criteria and 244 refused to participate. Verbal consent and baseline data was obtained from 417 participants. At the 12 and 24 month follow-up, data from 325 (78%) and 289 (69%) people respectively were collected. The study sample was similar in age and gender as the people who did not participate in the study, however, the study participants were less severely injured [17]. This is consistent with the inclusion criteria (catastrophic injuries were excluded). Those who withdrew during the study had similar sample characteristics as those who completed all questionnaires, except that the attrition group was on average 5 years younger, t (415) = 3.15, p = .002. The sample characteristics are displayed in Table 1.

**Table 1:** **Sample characteristics (n = 417)**

**Variable**	**Subclass**	**Mean (Standard deviation) or Percentage**
Age (years)		45.5 (17.2)
Sex	Female	61%
Country of birth	Australia	66%
Education	Low/ Medium	74%
	High	26%
Socio-economic status	Lower (1–5)	42%
	Higher (6–10)	58%
Work status	Employed	62%
	Unemployed	38%
Pre-injury health status	Good - Excellent	93%
	Fair-Poor	7%
Pain catastrophising – high	2 months	21.3% (89 of n = 417)
	12 months	14.6% (61 of n = 324)
	24 months	11.0% (46 of n = 287)
Injury severity	Mild	77.6%
	moderate	13%
	Serious	6.5%
	Severe	2%
Type of injury	Whiplash	56%
Previous claim		31%
Lawyer engagement	12 months	35% (114 of n = 323)
	24 months	34% (98 of n = 288)
Insurance company	One insurer	54% participants
	Other insurers	46% participants
Claims management dissatisfaction	2 months	37% (152 of n = 407)
	12 months	35% (113 of n = 323)
	24 months	35% (94 of n = 266)
Claim settlement	2 months	13% (53 of n = 417)
	12 months	53% (220 of n = 417)
	24 months	70% (293 of n = 417)
Anxiety/depression (moderate + severe)	2 months	37% (156 of n = 417)
	12 months	39% (127 of n = 325)
	24 months	29% (82 of n = 282)

### Quantitative analysis

The multiple forward logistic regression analysis revealed that the final regression model at baseline consisted of poor pre-injury health (Adjusted Odds Ratio [AOR] = 5.01, p = .002), high catastrophising (AOR = 3.79, p < .001), and claims management dissatisfaction (AOR = 1.88, p = .006). At 12 months, the independent predictors for anxiety were high catastrophising (AOR = 14.92, p < .001), and lawyer engagement (AOR = 3.08, p < .001). At 24 months, the factors that were significantly associated with anxiety were female gender (AOR = 2.51, p = .012), high catastrophising (AOR = 11.75, p < .001) and claims management dissatisfaction (AOR = 2.67, p = .004). The statistics including 95% confidence intervals (CI) of the significant factors are displayed in Table 2.

**Table 2:** **Results of multiple logistic regression analyses showing significant independent predictors of anxiety/depressive mood**

		**Anxiety/depression**	
	**2 months**	**12 months**	**24 months**
**Independent variables** ^**#**^	**AOR (95% CI)**	**AOR (95% CI)**	**AOR (95% CI)**
Gender (male – female)	-	-	2.51 (1.22 - 5.15)
Pre-injury health status (good – poor)	5.01 (1.82 - 13.77)	-	-
Catastrophising (low – high)	3.79 (2.25 - 6.38)	14.92 (6.34 - 35.10)	11.75 (4.97 - 27.78)
Lawyer engagement (no – yes)	-	3.08 (1.77 - 5.36)	-
Claims management (satisfied – dissatisfied)	1.88 (1.20 - 2.95)	-	2.67 (1.37 - 5.20)
Nagelkerke R^2^	.153	.343	.334

^#^At baseline, 14 predictors were entered into the regression. At 12 and 24 months, 15 variables were inserted (including lawyer engagement).

Table shows only significant variables (p < .05).

### Qualitative analysis

The qualitative analysis of unsatisfied remarks about the claims management revealed five themes: communication, determining compensation, medical treatment and assessment, paperwork, and liability assessment. A summary overview is provided in Table 3.

**Table 3:** **Problems experienced during the compensation process**

**Label**	**Problem**
Communication	Never returned calls, no initiative from the insurer side
	Lack of information
Determining compensation	Exceeding the $5000 limit of the no-fault claim
	Not being (sufficiently) compensated for costs
	Having to prepay costs and then seek reimbursement
	Waiting for loss of wages to be paid
	Claim settlement taking too long or feeling pressured to settle
Medical treatment and assessment	Reimbursement for treatment not being approved
	Waiting for treatment approval
	Discussing causality/having to provide entire medical history
	Having to undergo numerous medical assessments
Paperwork	Too much, too difficult, time consuming, repetitive
Liability assessment	Discussions about who was at fault

#### Communication

Many participants mentioned a lack of communication, such as claims managers who were hard to contact or who never returned calls. One participant had not been able to make contact, but nevertheless the claim manager stated the case would be closed. Another person received ‘only one letter, not even one phone call’, and that letter was ‘inadequate and impersonal’. Several complained that they constantly had to initiate contact, or that it took more than two weeks after claim lodgement before the insurer rang. One said he felt like he was ‘sending receipts off into the Bermuda Triangle’ and was unsure of whether anything would come of it.

Participants also mentioned a lack of information. They did not know what to do or how to go about making a claim. One participant did not realise that she had any entitlements. Two participants received a payment but had no idea to what this was related. Another participant was confused because the rehabilitation case manager had said something different from the claims manager about the number of treatment modalities she could claim. One participant said the communication worsened because of the involvement of a solicitor, because the insurance company then refused to deal with her directly.

#### Determining compensation

Several participants who lodged a capped claim Accident Notification Form were worried about exceeding the limit, particularly in case of loss of income. Some participants suffered major financial problems because they were not compensated for their loss of wages (e.g. could no longer pay their mortgage or had to sell their company). Participants were also frustrated with the time it took to get reimbursed, for example, re-imbursement of medical expenses often took a couple of weeks. Because loss of wages is compensated in the form of a lump sum award at settlement of the claim, it often took a long time for claimants to be recompensed. As a consequence, many participants stated they had to borrow money to meet household bills, such as for electricity costs. One person had had to move back home with her parents. Another one could not get the preferred treatment because she did not have the money to pay up front for the therapy. However, this was not always the fault of the insurance companies, as sometimes the medical practitioners would not bill the insurer directly, possibly because the insurer would not meet his or her actual charges.

Claims settlement evoked opposite responses: one felt ‘exhausted with the total run around’, he said he had ‘lived and breathed the process for the last two years and just wanted to put the whole thing behind him and try to get on with his life’. However, several other participants felt they were pressured to settle their insurance claim (e.g. they were ‘on the phone three times a day’): but they were worried they needed more treatment (for example, for on-going pain), which would no longer be paid for by the insurer after settlement.

#### Medical treatment and assessment

First, several participants mentioned that the insurance company did not approve reimbursement of a treatment or a scan, which their doctor had recommended. Sometimes reimbursement of a certain type of treatment, such as remedial, massage, or acupuncture, was refused without explaining why. Another participant stated that he was not allowed to change his general practitioner or physiotherapist. A second medical issue was the long waiting time for treatment approval, that is, a couple of weeks, during which the injured person was not having treatment. This waiting time sometimes had serious consequences; for example, one participant said that when the CT scan was finally approved, she found out she should not have been having physiotherapy treatment. Third, several participants had arguments about causality issues, for example, whether the injury was caused by the MVC or whether it was already pre-existing. Two participants were stressed because they had to release their entire medical history, which they considered to be unfair because that would mean that the insurer would also read private information such as gynaecological history and breast checks, which they said was not relevant to the claim. Finally, one participant mentioned the frequency and times of the day of medical assessments (e.g. ‘4 pm in the afternoon in the city’) to be unreasonable.

#### Paperwork

Many unsatisfied participants complained about the ‘mountains of paperwork’ that contributed to their stress. ‘Forms duplicated themselves. I had to give the same information multiple times’. ‘They are asking for three years’ worth of tax returns and three years’ worth of pay slips’. One of the issues raised was that the paperwork came at a time when participants were least able to cope, being in pain and recovering from injury. One participant had the funeral of her husband to deal with and therefore almost missed the deadline for lodging the claim. Another said that ‘everything has been made twice as bad as it was, having to get something faxed, having to get something copied, do this, do that’. One gave up because of the paperwork. It was time consuming, ‘spending half a day on the phone mostly on hold when feeling really unwell with a roaring headache’. Participants also complained that there was no assistance.

#### Liability assessment

A few participants had a dispute with the insurance company over who was at fault in the crash. The dispute usually centred on the fact that the other party involved in the crash had a different story about the circumstances under which the crash happened. Several injured persons were highly anxious and emotionally upset because they believed that the insurance company was trying to not pay their claim because liability was disputed. Some were confronted by a private investigator, which they found humiliating and degrading. One stated that somebody came to her house wanting to see the bike she was riding when the accident happened, asking personal and unrelated questions and she was made to feel as though it was totally her fault and that she was trying to get something to which she was not entitled.

## Discussion

The first aim of this study was to replicate the finding that a stressful interaction with the insurance company is associated with anxiety [8,10]. The current study indeed confirmed this, showing that dissatisfaction with the claims management process was associated with increased anxiety at 2 and 24 months after injury. However, dissatisfaction with the insurance company was not the best predictor because pain related catastrophising was stronger. In the literature, catastrophising has been discussed as a cognitive component active in depression and elevated anxiety [24]. However, the finding is a noticeable result *in the current context*, because pain-related catastrophising has not been taken into account in prior compensation and health studies. This may have led researchers to over-estimate the impact of other factors on the injured claimants’ well-being, and to disregard the importance of cognitions. It is often thought that claimants’ *coping* style is important in recovery. Our finding suggests it is worthwhile further investigating whether this is the case.

Three variables were associated with anxiety/depressive mood at one measurement only. Pre-injury health was associated with anxiety/depressive mood at 2 months after injury, which is consistent with a previous finding of a study among claimants in a no-fault scheme in New Zealand [25] and a study examining a general injury population after a motor vehicle accident in Victoria, Australia [10]. Lawyer engagement was associated with anxiety at 12 months after injury, which confirms previous findings [9,26]. An explanation could be that the claims procedure becomes more adversarial when a lawyer gets involved [27]. It could also be the case that a complex and/or confusing claims process contributes to anxiety, which encourages claimants to seek legal advice [12]. Finally, women were more likely to report problems with respect to anxiety/depressive mood 24 months after injury, which has previously been shown in other MVC studies at 12 and 36 months [28,29]. It is unknown why women have a greater chance of developing anxiety after trauma [29].

The current findings can be discussed in relation to the three explanatory theories described in the Introduction. First, the association between dissatisfaction with the insurance company, lawyer engagement, and the presence of anxiety/depressive mood may support the theory of secondary victimisation, because these were all claim factors associated with stress. Second, the factor ‘claim settlement’ was not associated with anxiety, so the current study does not support that claim settlement improves recovery (which would suggest secondary gain) [6]. In contrast, our findings endorse the review by Mendelson, who concluded that claim settlement does not have an effect on the recovery outcome [30]. Third, the significant associations found between anxiety/depressive mood, high catastrophising, and poorer pre-injury health could be relevant with respect to the theory that claimants have different characteristics compared to non-claimants and that these group differences are responsible for poorer recovery. However, to be able to draw a conclusion about the latter, a comparison between claimants and non-claimants is required; unfortunately, the current study included claimants only. There is a large prospective cohort study in progress that investigates this issue (trial registration number ACTRN 12613000889752).

The results should be interpreted with caution because of limitations. First, a selection or attrition bias could have been present. For example, there was an under representation of participants with limited education (2%), an over-representation of one insurance company compared to the market share, and those who withdrew from the study were significantly younger than the completers. Secondly, only a self-assessed, one-item subscale for the presence of anxiety/depressive mood was used, whereas a clinically administered questionnaire may have yielded more sensitive results. Furthermore, it is important to note that the current study consisted of an observational study design, and some confidence intervals are wide, so one should be careful not to draw conclusions about causality. Finally, not all measures that are possibly of influence to anxiety and depression, such as social support, were taken into account.

The second primary aim of the study was to (qualitatively) explore what is stressful about the interaction with the insurance company. Five themes were found: communication, determining compensation, medical treatment and assessment, paperwork, and liability assessment. Lack of communication, problematic treatment approvals, and the burden of delayed claims settlement, were themes that stood out as stressful and burdening. These results were also addressed as problematic in previous studies about the car injury compensation process [11,12]. Also noticeable was the reported financial burden that is associated with delayed payment, especially in case of loss of earnings, which, in fault-based schemes, is often compensated in the form of a lump sum award at claims settlement. Since claims settlement can take 12 or 24 months, a lack of income highly stressed some injured participants for a considerable length of time. The impact of delayed or interrupted payments has been reported in the workers’ compensation literature [14,31], and administrative delays were found to be positively associated with the odds of developing chronic disability [32], indicating that this is a serious issue. A specific suggestion to relieve the stress of financial uncertainty is to introduce payments for loss of income after a qualifying period rather than by reimbursement at settlement only.

The problems with proving liability and causality were not discussed in qualitative studies previously, although they have been pointed out as risk factors potentially resulting in elevated stress in a literature overview [2]. The extra stress seems to be preventable with better explanations as to why certain questions are being asked, faster treatment approval times and pre-payment for selected evidenced based treatments. Finally, the burden of too much paperwork has been discussed in workers’ compensation settings [14,31], although it might be a more prominent problem in the current setting, that is, a fault-based compensation scheme, whereas the previous workers’ compensation studies examined a no-fault scheme, in which people are eligible for compensation regardless of fault. It seems worthwhile to review the number and length of these forms, as well as to simplify questions asked, prevent duplicity and provide assistance with form completion. In NSW, the forms are currently being reviewed. Overall, it is concluded that many of the findings have already been discussed in other studies in different parts of the world describing either car injury or workers’ compensation schemes. The fact that these topics are recurrent topics described in a growing body of literature from different schemes all over the world shows the importance of the problem and the need for change.

The qualitative research has limitations. First of all, qualitative research, in general, is difficult to generalise, because the analysis focuses on individuals rather than group samples [23]. Qualitative research cannot provide information about the frequency, or overall importance, of specific issues. A strength of this present study, however, is the large sample size, improving the generalisability of the findings. Secondly, the analyses deal with the perceptions of people who have made claims and therefore are not independently verifiable. Verification of the overall findings with the Motor Accidents Authority revealed that, for example, approval of treatments also needs to be in accordance with clinical practice guidelines, the time of day for medical assessments is not determined by the insurer but by the medical assessor, and that an independent claims advisory service is available to provide practical advice and assistance without cost to the injured person. Regarding the communication issues, claim managers officially have 10 working days to reply to requests, which is ‘a couple of weeks’, so that means the participant’s claims manager seems to have acted according to the insurance legislation and regulations [16]. Similarly, common practice restricts claims managers from directly communicating with a legally represented claimant. Finally, it should be noted that the participants were involved in a hybrid, mainly fault-based compensation scheme [33]. Most countries have a fault-based compensation scheme for injury after a traffic crash, but some countries or states have a no-fault scheme. One should be careful to generalise these findings to a no-fault setting, because fault-based schemes are hypothesised to be more adversarial.

## Conclusions

Two conclusions can be drawn from this study. First, it was found that pain-related catastrophising is strong predictor of anxiety/depressive mood in people injured in MVC. This means that it could be beneficial to assess the injured person’s level of catastrophising in order to optimize treatment and to improve well-being. People who score highly on catastrophising may benefit from social or professional support addressing feelings of helplessness and hopelessness, and encouraging a problem-solving adaptive coping style approach with respect to their injury, pain and claim management process.

Second, this study found an association between claims management dissatisfaction and the presence of elevated anxiety/depressive mood. Stress was associated with problems of communication, medical treatment, and claim settlement. Overall, it seems that most problems reported by the participants could be mitigated if claim managers would adopt the attitude that they are the ‘problem owner’ and become more proactive. It is acknowledged that ‘improving communication’ is already on the agenda of many insurers, but ‘problem ownership’ is more than that. It is about taking responsibility for the fact that damage has been inflicted that now has to be mitigated, assessed and compensated. This can be achieved by a proactive form of claim management (e.g. taking the initiative in the interaction, frequent updates about the state of affairs, smooth approval of treatment, expedient reimbursement of incurred costs, and adequate interim payments of established compensable loss of income). Such an attitude could potentially restore the injured person’s feelings of injustice that harm has been inflicted to him/her [34]. A previous study showed that a proactive approach by the insurance company facilitates the claimants’ return-to-usual-activities [35]. This suggests that proactivity would not only be beneficial for injured people but also may imply a cost reduction for insurance companies.
